# Disrupting the Photochemical Landscape of a β‑Diketone via Electrostatic Perturbation of Ground-State Tautomers

**DOI:** 10.1021/jacs.5c12521

**Published:** 2025-12-02

**Authors:** Cate S. Anstöter, Sarah A. Wilson, Natalie G. K. Wong, Giel Berden, Jos Oomens, Anouk M. Rijs, Caroline E. H. Dessent

**Affiliations:** a Department of Chemistry, 8748 University of York, Heslington, York YO10 5DD, U.K.; b School of Chemistry, 150842 University of Glasgow, Joseph Black Building, University Ave., Glasgow G12 8QQ, U.K.; c FELIX Laboratory, Institute for Molecules and Materials, 6029 Radboud University, Toernooiveld 7, 6525 ED Nijmegen, The Netherlands; d Division of Bioanalytical Chemistry, Department of Chemistry and Pharmaceutical Sciences, AIMMS Amsterdam Institute of Molecular and Life Sciences, 1190 Vrije Universiteit Amsterdam, 1081 HV Amsterdam, The Netherlands; e Centre for Analytical Sciences Amsterdam (CASA), Amsterdam, The Netherlands

## Abstract

Photochemically triggered control of keto–enol equilibria is of growing importance in rational synthetic strategies. Questions exist, however, about the extent to which the electrostatic effects (i.e., interactions with counterions and solvent) can perturb or direct such photocatalytic approaches. Here, we directly address the question of whether electrostatic tuning via alkali metal binding can affect the photochemistry of the classic β-diketone molecule, avobenzone, which exists in keto and enol forms. A combination of photodissociation mass spectrometry over the ultraviolet (UV) (390–238 nm) and infrared (IR) (800–1800 cm^–1^) ranges and quantum chemical calculations (density functional theory (DFT) and SCS-ADC(2)) is applied to isolated (gas-phase) alkali metal-avobenzone complexes for the first time. We find that Na^+^, K^+^, and Rb^+^ binding only modestly perturbs the avobenzone π*-character excited states compared to the bare molecule. However, electrostatic binding has a dramatic overall effect since cation binding reverses the relative stability of the tautomers on the electronic ground state: without cation binding, the enol dominates, whereas upon cation binding, the keto form dominates. This is of critical photochemical importance as the ketone tautomer is the doorway geometry to photoinstability through providing access to optically dark triplet states that are adjacent to bright singlet states. Ion binding therefore opens a route to enhanced photochemical activity and molecular dissociation. Our results demonstrate a paradigm where electrostatic binding, here with a metal cation, perturbs the molecular keto–enol photochemistry through disruption of the ground-state electronic surface.

## Graphical abstract

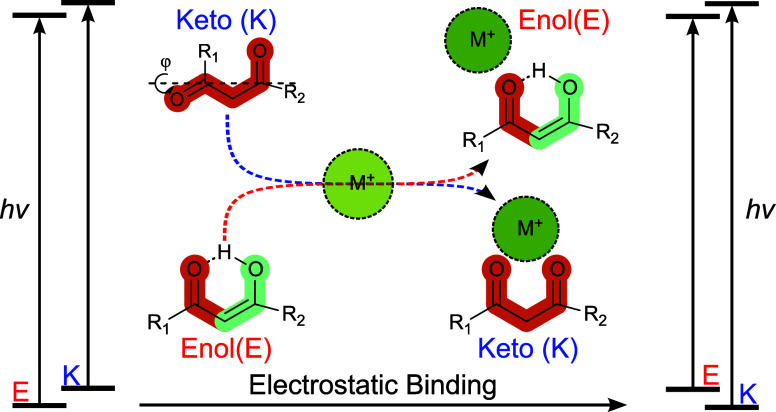

## I Introduction

Keto–enol tautomerism is a basic mechanistic phenomenon that underpins much of organic chemistry.^1^ While keto–enol equilibration typically involves acid and base catalysis in thermal protocols,^2,3^ it can also be triggered by light.^4^ Such photochemically driven keto–enol tautomerization has become increasingly important in synthetic chemistry, particularly in the production of complex chemotypes and pharmaceuticals.^5^ A specific example is provided by the use of photoenolization in cycloadditions with dienophiles to form cycloadducts,^2,6,7^ which is now a powerful and widely used tool in asymmetric synthesis.^8^

As controlled photochemical strategies have gained increasing importance across synthetic chemistry, awareness has been growing that intramolecular photochemical processes can be influenced by the local chemical environment. An elegant example was demonstrated by Venkatraman and co-workers, who showed that hydrogen-bonding interactions with a solvent environment could dramatically alter the photophysical properties of benzophenone,^9^ a prototypical photocatalyst. Similarly, a small number of recent studies have shown that metal ion coordination can impact on photophysical properties. Marlton *et al*. demonstrated that it is possible to electrostatically tune the key ^1^ππ*-^3^nπ* energy gap of the photoinitiator, Irgacure, via selective cation binding and hence its dissociation propensity.^10^ Robertson *et al*. subsequently showed that Mg^2+^ ions perturb the S_2_ electronic state of acetophenone, another photoinitiator, affecting both intersystem crossing and internal conversion rates.^11^

These recent examples of metal-ion-tuned photochemistry motivate questions of whether similar intermolecular interactions could affect keto–enol tautomerization in a way that might enhance photosynthetic strategies, either through the preparation of higher-energy reactive enol isomers or through the enhancement of photochemically driven dissociation. We focus here on directly probing whether electrostatic complexation by alkali metal cations can affect the photochemical properties of a classic, keto–enol β-diketone tautomer molecule, avobenzone. Our choice of avobenzone (AVB) is motivated by the fact that it is known to undergo photodissociation following excited-state tautomerization, providing a ready diagnostic for us to track excited-state properties.^12–14^ We use laser photodissociation mass spectrometry as a novel photochemistry technique,^1,15–23^ to select and probe the isolated metal ion-avobenzone complexes M^+^·AVB, where M = Na, K and Rb, in the gas-phase. The complexes are then subjected to wavelength-dependent UV laser photodissociation (390–238 nm), allowing the measurement of the gas-phase absorption (photodepletion) spectra and photodissociation products (Section S1). Importantly, this approach allow us to directly detect photochemical products across the entire photoexcitation range,^15–17^ and hence assess the extent to which differential cation binding influences molecular dissociation. As a precursor to the UV photochemistry measurements, we present infrared multiple-photon dissociation (IRMPD) spectroscopy over the 800–1800 cm^–1^ range of the M^+^·AVB complexes to characterize the alkali metal binding motifs.^24,25^ The experiments are supported by advanced quantum chemical calculations (SCS-ADC(2))^26^ to provide detailed insight into the effect of metal cation binding on the keto–enol photochemistry.

Aside from being a classic β-diketone, avobenzone (AVB: 4-*tert*-butyl-4′-methoxy dibenzoylmethane; BD-DBM or Parsol 1789) is a widely used UVA sunscreen molecule.^27–30^ It is also of interest as a member of a family of molecules known to take multiple isomerization pathways following UV excitation.^31–34^ The key tautomeric forms of AVB involved in this photoisomerization are illustrated in Scheme 1. Photoexcitation of AVB is believed to lead to nonadiabatic population of high-lying S_0_ vibrational levels, which can lead to isomerization to a less photostable form.^35,36^ Previous photolysis experiments performed in hexane have shown that AVB photodegradation occurs by a Norrish type 1 mechanism.^37,38^ In the ground state, AVB primarily exists in its chelated enol (E) form due to the stabilizing intramolecular hydrogen bond, although the (di)­keto (K) form is typically also present at lower populations. The E-tautomer is the active UVA chromophore (λ_max_ = 355 nm), with photoisomerization leading to nonchelate forms, which gradually populate the keto-form. The K-tautomer absorbs at higher UV energies (λ_max_ = 265 nm) and is believed to be responsible for formation of a reactive triplet.^38–41^ Wang *et al*., for example, demonstrated that AVB photodegrades (in the UVA) more quickly as a function of chlorine substitution at the α-carbon (C2) since this promotes the K-tautomer population.^42^

**Scheme 1:**
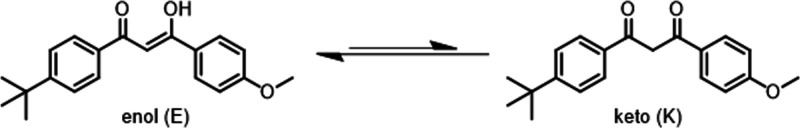
Schematic Diagram of the Chelated Enol (E) and (Di)­keto (K) Tautomers of Avobenzone (AVB)

The effect of solvents on AVB isomerization, and hence its photostability, have been explored qualitatively in previous work.^29,43,44^ These studies suggest that other local environmental factors may also influence the extent of AVB tautomerization and photoproduct formation; a phenomenon that is now directly addressed in the work presented here through applying the combination of quantum chemistry and gas-phase spectroscopy. Importantly, applying UV laser photodissociation mass spectrometry to M^+^·AVB allows us to selectively investigate the photochemistry of the distinct keto- and enol-forms, since the tautomers absorb strongly over different ranges of the UV.^12^ Overall, this study aims to determine the extent to which alkali metal complexation is able to tune photoactivated keto–enol tautomerisation, and hence provide a broader understanding of the potential of metal salts as electrostatic control agents in photochemical synthetic processes.

## III Results and Discussion

### Structures

Quantum-chemical calculations (B3LYP/cc-pVTZ) of the M^+^·AVB complexes resulted in four distinct low-energy structures associated with four possible tautomers, which are illustrated schematically in Scheme 2. The relative energies for the various isomeric complexes of each of the M^+^·AVB systems are given in Table S2.1. (Section S2 provides results for additional calculations performed at different levels of theory; these agree well with the B3LYP/cc-pVTZ results). Calculations at the B3LYP level have previously been shown to provide vibrational frequencies that are in good agreement with IRMPD spectra for alkali metal cation containing complexes.^45,46^

**Scheme 2:**
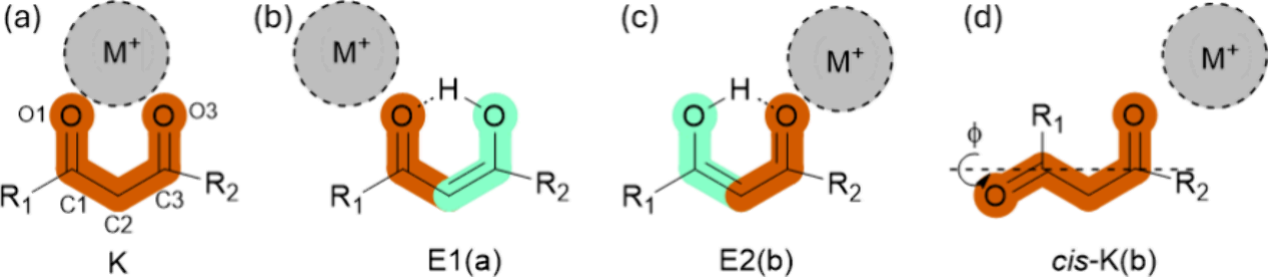
Coordination Motifs for the M^+^·AVB (M^+^ = Na^+^, K^+^, and Rb^+^) Complexes^a^

a (a) Bidentate binding to the K tautomer, (b) monodentate binding to O1 of the E1­(a) tautomer, (c) monodentate binding to the O3 of the E2­(b) tautomer, and (d) monodentate binding to the O3 of the cis-K tautomer. Keto functional groups are highlighted in orange, with enol groups in green. Φ represents the dihedral angle between O1–C1–C3–O3 about the dotted axis, with R1 = Ph­(t-Bu) and R2 = Ph­(OMe). Atomic numbering follows Kojić *et al.* ^52^

The preferred alkali metal ion binding site is a carbonyl group for all of the stable complexes. This binding preference has been observed previously in other studies of alkali metal cation-molecule complexes, particularly for smaller cations.^34,47–51^ The K-isomers are nonplanar while the E-isomers are planar as the C1–C2 and C2–C3 bonds have double-bond character. E1 and E2 represent two distinctive E-tautomers, with the hydroxyl group on the C3 and C1 positions, respectively. An E1­(b) type structure also exists (Table S2.1), with the enol hydroxyl on the C3 position, with the cation binding to the O3.

All of the M^+^·AVB metal ion complexes have the K-isomer as the global-minimum structure (Table S2.1), due the cation preference for carbonyl binding. In the K-isomers, the alkali metal cation is able to engage in two favorable O-M^+^ intermolecular interactions, whereas the E-geometry only allows for a single interaction between the metal cation and an electronegative oxygen atom. While the K-isomers are the dominant lowest-energy structures for all of the M^+^·AVB complexes, the energy gap between the K and E-tautomers lowers as the cation size grows, due to the increased steric repulsion present for the larger cations in the more constrained K-geometry, as well as the lower ion-dipole interaction for the larger cation.^53^ (K-tautomers:E-tautomers relative populations for Na^+^·AVB, K^+^·AVB, and Rb^+^·AVB are 100:00, 99:1, and 98:2, respectively: see Table S2.1)

### IRMPD Spectroscopy

Figure 1 displays the IRMPD total ion yield spectra of the M^+^·AVB complexes. (Spectral intensities were calculated via depletion measurements: see Section S1b) The spectra display several common features, including a relatively narrow band at ∼1670 cm^–1^, a broad band around 1580 cm^–1^, likely due to at least two overlapping vibrations, another broad band between 1280 and 1350 cm^–1^, which again is likely to include a number of overlapping vibrations, and the most intense and relatively narrower band at 1170 cm^–1^. There are additional low-intensity vibrational features across the range from 1400 to 1550 cm^–1^ which are more distinct on going from Na^+^·AVB to Rb^+^·AVB.

**Figure 1:**
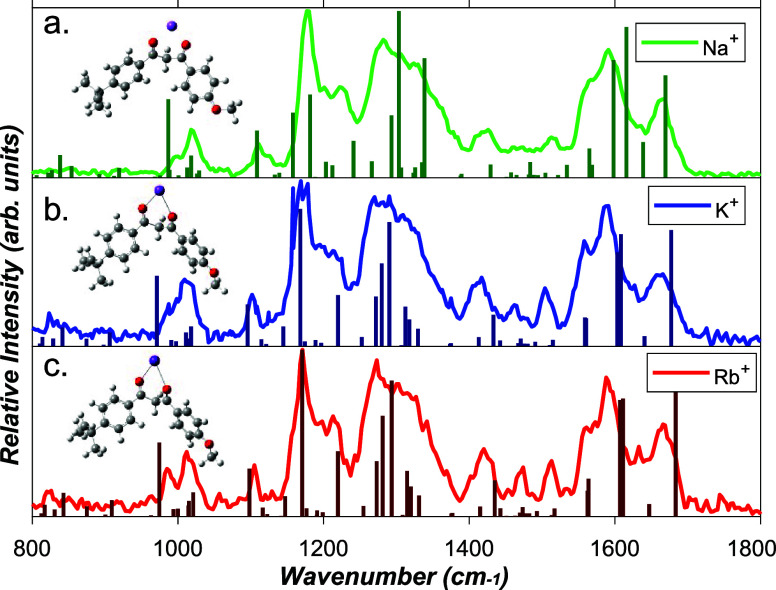
IRMPD spectra of (a) Na^+^·AVB (green), (b) K^+^·AVB (blue), and (c) Rb^+^·AVB (red), overlaid with simulated IR stick spectra obtained for the B3LYP/cc-pVDZ optimized keto-structures shown as inserts. Simulated spectra have been scaled by 0.97 following refs 54 and 55 .

IR spectra for K and E isomers obtained from the calculated M^+^·AVB structures are shown in the figures of Section S3. Comparison of the experimental and calculated spectra reveal that the experimental spectra are most consistent with K isomers. This assignment is based primarily on the K isomer calculations predicting a symmetric diketone carbonyl stretch around 1660 cm^–1^, a mode that is unique to the K isomers. We assign the experimental vibrational features at 1666 (Na^+^·AVB), 1666 (K^+^·AVB), and 1668 (Rb^+^·AVB) cm^–1^ to this diketone symmetric stretch mode. (This mode is calculated to occur at 1649, 1652, and 1656 cm^–1^ for Na^+^·AVB, K^+^·AVB and Rb^+^·AVB, respectively at the B3LYP/cc-pVTZ level.) Importantly, this vibration is predicted by the calculations to occur approximately 50 cm^–1^ above a more intense pair of vibrations associated with symmetric and asymmetric stretches of the aromatic C–C bonds. This prediction fits well with the experimental spectra and the occurrence of the broad (double-vibration) feature which peaks at 1591 and 1566 cm^–1^. The other notable features of the experimental spectra also fit well with the calculated K-isomer structures, as discussed in Section S3. In contrast, the calculated spectra of the E-isomers do not agree well with the experimental spectra. The key distinctive vibrational mode for the E-isomers is an in-plane bend of the enol OH group, which is predicted to occur as a strong mode at ∼1450 cm^–1^ but not replicated in the experimental spectra. We conclude that the K-isomer is the dominant tautomer present in the experimental ion ensemble for all complexes. The assignment of the IRMPD spectra is discussed in further detail in Section S3.

### UV–Vis Spectroscopy

The gas-phase photodepletion (PD) spectra of the M^+^·AVB complexes across the range from 3.2 to 5.2 eV are displayed on Figure 2a. These action spectra provide insight into the M^+^·AVB excited states, since they relax with molecular fragmentation and hence depletion of the complex.^56^

**Figure 2:**
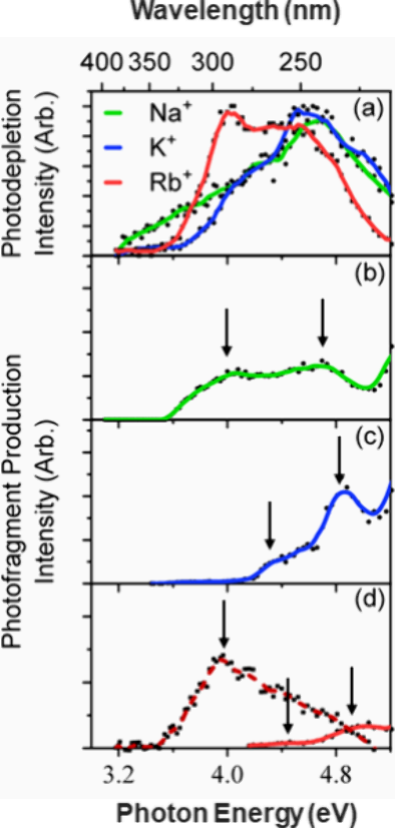
(a) Photodepletion (PD) spectra of M^+^·AVB (M^+^= Na^+^, K^+^, Rb^+^), and associated UV summed photofragment (PF) action spectra of (b) Na^+^·AVB, (c) K^+^·AVB, and (d) Rb^+^·AVB with summed PFs *m*/*z* 234 + 260 + 380 (solid) and Rb^+^ loss (*m*/*z* 85) (dashed). The arrows indicate locations of the assigned experimental bright excited states.

All three complexes display intense, broad absorptions across the UVA-UVB region as expected for the chromophore AVB,^26^ with spectral profiles that are consistent with the presence of at least two distinct but overlapping absorption bands. For Na^+^·AVB and K^+^·AVB, the absorption spectra both display relatively gentle absorption onsets above 3.2 eV, that flatten around 4.20 eV. These very broad, lower-energy absorptions are then followed by a further rise in absorption intensity that peaks at 4.7 and 4.6 eV, respectively. The Rb^+^·AVB PD spectrum has a similar profile, but displays relatively more intense photodepletion in the lower-energy region (<4.0 eV). All three PD spectra decrease in intensity toward the high-energy spectral region, although this decrease is more distinct for Rb^+^·AVB given that its spectrum is red-shifted compared to the Na^+^·AVB and K^+^·AVB spectra.

### Photofragmentation of M^+^·AVB

Figure 2b–d present the photofragment (PF) production spectra of the M^+^·AVB complexes. The PF action spectra are valuable to support the assignment of the locations of the excited states present in the PD spectra, since they contain less spectral congestion.^57,58^

The PFs observed upon photoexcitation of the three M^+^·AVB complexes correspond to the pathways listed below. (Chemical structures assigned to the PFs are given in Section S4.) The PF production pathways correspond to either Norrish Type 1 α-cleavage (the known photodissociation pathway for AVB in solution);^12,36^

$$M^{+}\cdot \mathrm{AVB}+h\nu \to C_{11}H_{13}O+m/z(149+M)$$ 1a

$$\to C_{8}H_{7}O_{2}+m/z\left(175+M\right)$$ 1b

To α-cleavage of AVB accompanied by homolytic CH_3_–O dissociation and methyl loss;^59^

$$M^{+}\cdot \mathrm{AVB}+h\nu \to C_{19}H_{19}O_{3}+m/z(295+M)$$ 2

Or by alkali metal cation loss from the complex:^60^

$$\to C_{20}H_{22}O_{3}+m/z\left(M\right)$$ 3

Figure 2b displays the PF action spectrum of Na^+^·AVB, plotted as a sum of all the observed PFs. (Fragmentation pathways [Disp-formula eq1a], 1b, and 2 are present.) Peaks in PF production are clearly visible at 4.00 and 4.70 eV (arrows on Figure 2b), corresponding to the peaks of the experimental excited states. PF production increases smoothly into the UVC due to the increasing internal energy following photoexcitation.^16^

The summed PF action spectrum for K^+^·AVB is displayed in Figure 2c, with peaks in PF production being visible at 4.30 and 4.90 eV. (Fragmentation pathways [Disp-formula eq1a], 1b, and 2 are present.) The excited-state locations are marked by arrows on the PF spectrum. PF production again increases smoothly into the UVC.

Finally, Figure 2d displays the PF action spectrum for Rb^+^·AVB. The solid line spectrum with an onset around 4.15 eV corresponds to the summed PFs associated with pathways [Disp-formula eq1a], 1b, and 2) associated with rupture of the AVB molecule. This action spectrum is directly comparable to those shown for Na^+^·AVB and K^+^·AVB in Figure 2b,c. The dashed line spectrum which displays an onset at 3.5 eV corresponds to photoproduction of the Rb^+^ ion (*m*/*z* = 85). Peaks in PF production are visible at 4.45 and 4.90 eV for the summed PFs associated with rupture of AVB (solid line spectrum), while production of Rb^+^ peaks at 3.95 eV. These excited-state locations are indicated by the arrows on Figure 2d. Table 1 summarizes the locations of the bright states for the M^+^·AVB complexes, and includes the calculated vertical excitation energies (VEEs) which are discussed in the next section.

**Table 1:** Energies (±0.05 eV) of the Experimentally Observed Optically Bright States (eV) for the M^+^·AVB Complexes Obtained from Their PD (Figure 2a) and PF Action Spectra (Figure 2b–d), along with Calculated Vertical Excitation Energies (VEEs) in Parentheses^a^

	Na^+^·AVB	K^+^·AVB	Rb^+^·AVB
Exp λ_max‑1_	–(3.63)	–(3.69)	3.95 (3.71)
Exp λ_max‑2_	4.00 (4.23)	4.30 (4.45)	4.45 (4.50)
Exp λ_max‑3_	4.70 (4.79)	4.90 (4.95)	4.90 (5.01)

a Table 2 provides further details on the vertical excitation energies (VEEs).

Rb^+^ is the only one of the alkali metals we directly detect as a PF, since the low-mass cutoff precludes the detection of Na^+^ and K^+^ PFs. Nonetheless, we can be confident that they are produced as PFs from their respective complexes since there is a distinct mismatch in the profiles of the Na^+^·AVB and K^+^·AVB PF action (Figure 2b,c) and PD spectra (Figure 2a), especially in the low-energy spectral range. Through comparison with the Rb^+^·AVB spectra, the PF action and PD spectral mismatches for Na^+^·AVB and K^+^·AVB can therefore be attributed to our inability to detect Na^+^ or K^+^. Some insight can be gained into the Na^+^ and K^+^ PF production profiles by inferring their action spectra as the difference between the PD spectra and the sum of the other PF spectra. It is clear from this analysis that photofragmentation into Na^+^ and K^+^ is the dominant pathway in the low-energy spectral regions, in particular below 3.6 eV for Na^+^·AVB and below 4.4 eV for K^+^·AVB. Section S5 provides further discussion of the PFs. Note that the fragmentation onsets are not controlled by thermodynamic factors, but rather by the positions of the bright electronic states which are discussed further in the next section. (Section S6 provides details of collision induced dissociation measurements for the M^+^·AVB complexes which do show the expected thermodynamic binding trends.)

### Electronic Excited States of the M^+^·AVB Complexes

Quantum chemical calculations were performed to assign the electronic spectra and provide further insight into the nature of the excited states (Section S6). Table 2 summarizes the vertical excitation energies (VEEs) and oscillator strengths of the key electronic transitions for the M^+^·AVB complexes in their K- and E-forms. Most notable is the fact that the single bright state present for the π-delocalized enol conformer (1ππ*) is broken into two comparable oscillator strength transitions (1ππ* and 2ππ*) in the ketone.

**Table 2:** SCS-ADC­(2) Calculated Vertical Excitation Energies (eV) and Oscillator Strengths over the Range of 3.2–5.2 eV for the Bright Electronic Transitions of the K- and E-Tautomers of the M^+^·AVB Complexes^a^

	Na^+^·AVB		K^+^·AVB		Rb^+^·AVB	
	ketone		ketone		ketone	
electronic transition	VEEs (eV)	oscillator strength	VEEs (eV)	oscillator strength	VEEs (eV)	oscillator strength
1ππ*	4.23	0.352	4.45	0.494	4.50	0.544
2ππ*	4.79	0.491	4.95	0.502	5.01	0.495
1nπ*	4.13	0.111	4.13	0.012	4.16	0.003
2nπ*	4.37	0.096	4.23	0.037	4.23	0.017

	enol		enol		enol	
electronic transition	VEEs (eV)	oscillator strength	VEEs (eV)	oscillator strength	VEEs (eV)	oscillator strength
1ππ*	3.63	1.110	3.69	1.087	3.71	1.082
2ππ*	4.83	0.105	4.74	0.101	4.74	0.122
1nπ*	4.54	0.046	4.41	0.011	4.37	0.010

a Section S6 provides a listing of additional electronic transitions.

Our prediction of the excited-state locations for the K-tautomers are in good, qualitative agreement with the experimentally observed excited states (Table 1). This is important as it supports our conclusions from the IRMPD spectra that the K-forms are the dominant tautomers present experimentally. Indeed, if E-tautomers were dominant, we would expect to see the most intense excitation occurring close to 3.7 eV experimentally, with considerably less excitation above 4 eV, which is evidently not the case for Na^+^·AVB and K^+^·AVB. For Rb^+^·AVB, enhanced lower-energy excitation is evident in both the PD and PF spectra peaking at around 3.95 eV, potentially indicating that a proportion of enol tautomers is present for this cluster, leading to a bright enol 1ππ* transition being observed. Our ground-state calculations indicate that E-tautomers are most likely to be present for Rb^+^·AVB, albeit still at significantly lower abundances than the K-tautomers. (K-tautomers:E-tautomers relative populations for Na^+^·AVB, K^+^·AVB, and Rb^+^·AVB are 100:00, 99:1, and 98:2, respectively. Table S2.1)

A qualitatively similar picture of the excited-state locations is evident for all three M^+^·AVB complexes, with only modest differences in the VEEs as the cation is varied (Section S7). This is particularly true for the keto-isomers. As an example, the VEEs for both the 1ππ* and 2ππ* states of the K-tautomer increase only modestly on going from Na^+^·AVB to K^+^·AVB to Rb^+^·AVB. This result contrasts with recent work on metal ion complexes of Irgacure where red shifts >1 eV were observed.^10^ The lack of strong perturbation of the VEEs as a function of the identity of the cation seen in the current work is consistent with the ion binding locations not being aligned with the transition dipole moments of the key bright transitions. For M^+^·AVB, therefore, we can conclude that changing the metal cation results in only minor perturbations of the relative locations of the excited states.

## IV Further Discussion

### Comparing the Photochemistry of Neutral AVB and Its Complexes with Metal Cations

To understand how alkali metal ion complexation affects the electronic structure of AVB, it is instructive to begin by exploring the properties of neutral AVB. Figure 3 displays the calculated minimum energy E- and K-conformers of AVB, illustrating that the E-minimum corresponds to a planar π-delocalized system whereas the K-minimum corresponds to a twisted form with the two central oxygen atoms rotated away from one another. Due to the stabilizing effect of the π-delocalization, the E-form is the global minimum structure (Table S2.1). This is a critical result, when compared to the M^+^·AVB systems, where the global minima are K-isomers. Therefore, complexation of an alkali metal cation to AVB flips the tautomeric energy ordering compared to the bare molecule, and reverses the keto–enol energy ordering.

**Figure 3:**
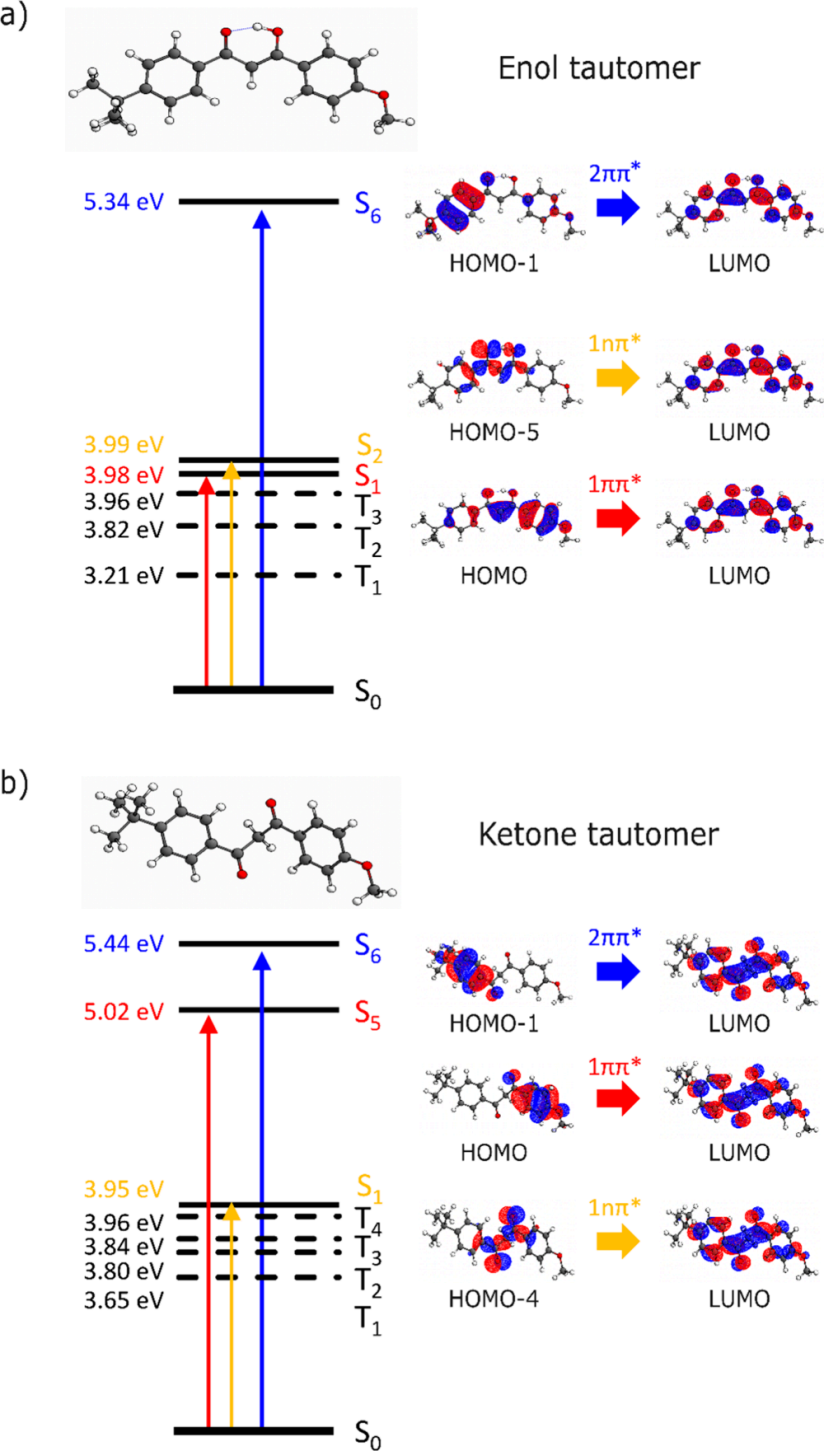
Bright electronic excitations for neutral AVB in its (a) enol and (b) ketone tautomeric forms, with the associated molecular orbitals for the transitions.

The key predicted bright electronic excitations and the associated molecular orbitals for neutral AVB, when no metal cation is present, are displayed on Figure 3 along with the VEEs and state assignments. (Section S6 provides details of additional states.) The first clear difference in the electronic structure of the two tautomers can be understood through inspection of the highest occupied molecular orbital (HOMO), which reveals the origin of the change in the 1ππ* VEEs for the two systems. The site of the enol-keto hydrogen in the tautomers dictates the extent of π-conjugation, with the E-form conjugation spreading over the bridge between the two aromatic rings. Contrastingly, when the enol hydrogen moves to the bridging carbon in the K-form, it breaks this conjugation yielding two detached aromatic chromophore units. Therefore, the HOMO of the E-tautomer extends across the two chromophore units, whereas in the K-form, it is localized to the anisole chromophore. The extended π-conjugation in the former leads to a lowering of the VEE of the 1ππ* state by ∼1 eV.

A second consequence of the different enol-keto hydrogen site relates to the character of the lowest occupied molecular orbital (LUMO). In the E-form the LUMO is antibonding across the central molecular bridge connecting the two aromatic rings, so that all bright (singlet) ππ* as well as the nπ* transitions are antibonding. However, for the K-tautomer the LUMO is bonding, and as such all of the ππ* and the nπ* transitions are bonding.

A similar overview of the bright transitions for Na^+^·AVB is displayed in Figure 4, as a representative example of the M^+^·AVB complexes, along with the minimum-energy tautomer structures. As discussed above, a geometry change occurs for the ketone-tautomer upon cation binding, with the two ketone groups now aligning so that each directly binds to the cation. However, the characters of the bright transitions for Na^+^·AVB are very similar to those of neutral AVB, with all of the transitions for the bright states of the E-tautomer being antibonding in character, while all of the transitions for the K-tautomer are bonding. The picture that emerges is that cation binding only modestly affects the excited state surfaces. Indeed, this is also evident in the fact that the calculated VEEs change only very modestly as the alkali metal cation changes (Table 2 and Section S7).

**Figure 4:**
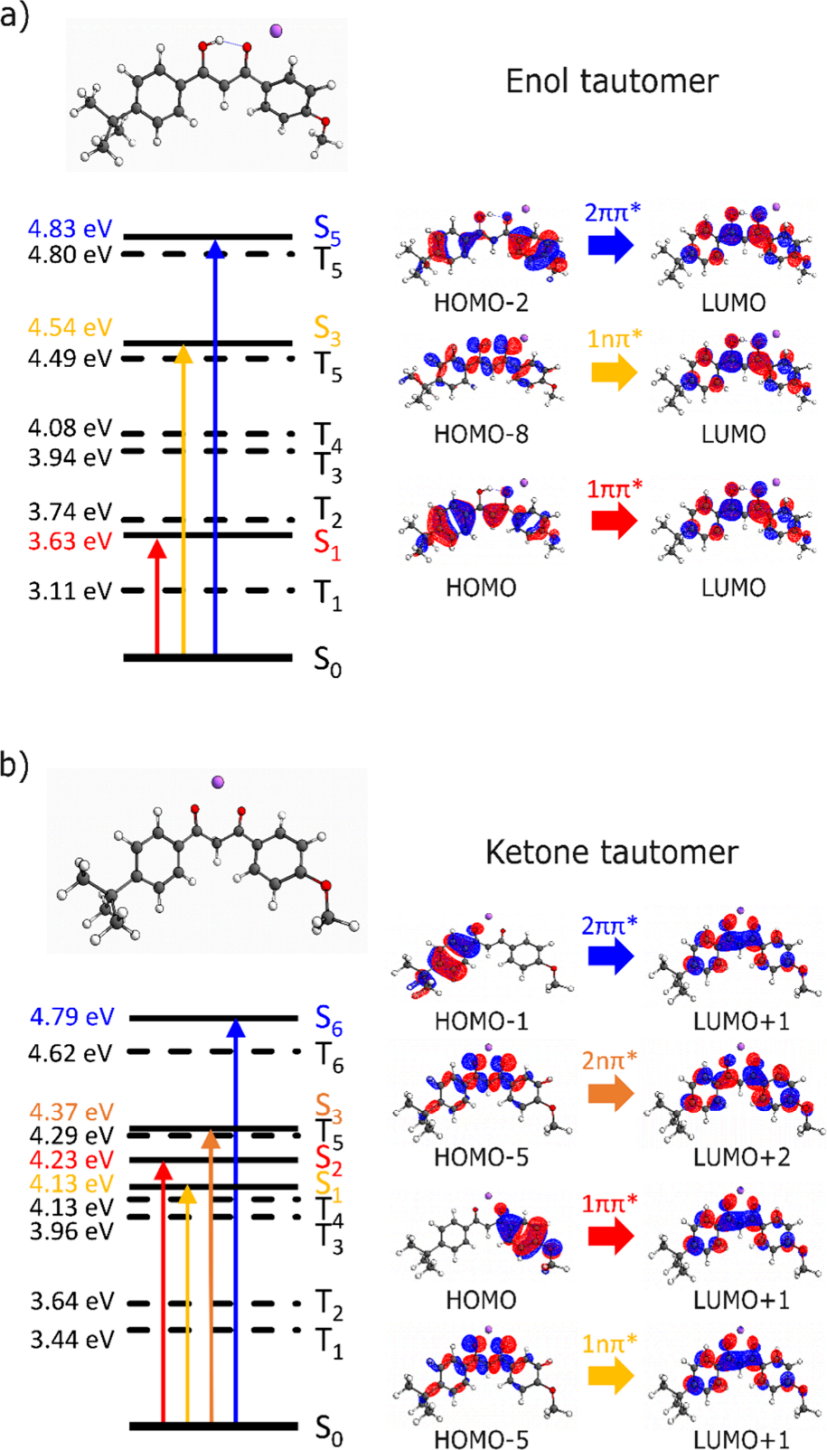
Bright electronic excitations for the Na^+^·AVB complex in its (a) enol and (b) ketone tautomers, with the associated molecular orbitals for the transitions.

The excited-state calculations performed here also provide insight into the nature of the photodissociation pathways observed experimentally. The dominant experimental fragmentation patterns for the M^+^·AVB complexes are consistent with Norrish Type 1 α-cleavage across the central molecular bridge between the two chromophoric units. This fragmentation pattern has previously been identified as the main photoinduced degradation pathway for neutral AVB and has been widely postulated to occur via optically dark triplet states.^12,36^ Inspection of the triplet states of the K-form of the M^+^·AVB complexes (Figure 4b) indicates that the triplet states in the vicinity of the bright singlet states are in fact antibonding across this region of the chromophore. This MO analysis of the character of the triplet states provides support that this state is the surface on which Norrish cleavage and the production of the fragments occurs. The triplet states of the E-form are roughly nonbonding in nature, adding further weight to our conclusion that the K-form of the M^+^·AVB complexes is the prevalent experimental form with respect to both the bright ππ* states observed and the identity of the photofragments produced.

## V Concluding Remarks

Through applying laser spectroscopy and quantum chemical calculations, we have investigated how metal cation binding perturbs the photochemistry of avobenzone as a model system for exploring electrostatic tuning of keto–enol photochemistry. We find that Na^+^, K^+^ and Rb^+^ binding only modestly perturbs the location and nature of the AVB excited-states and their associated photochemistry. However, of key importance is the fact that electrostatic binding flips the energy ordering of the ground-state E- and K-tautomers of AVB: For uncomplexed AVB, the E-tautomer is the global minimum structure, whereas cation complexation leads to the ketone becoming the dominant isomer. This is critical in the photochemical behavior of the system, since the K-tautomer has long been acknowledged to be the doorway structure to triplet excited states that are dissociative and hence lead to photodegradation of AVB. For neutral AVB, these ketone geometries are only accessed indirectly following initial UVA photoexcitation of the predominant E-structures,^38–41^ but upon complexation with an alkali metal, the K-forms will dominate the ground-state ensemble and facilitate photoexcitation to unstable dissociative pathways.

AVB is very widely employed as a UVA filter, including within many commercial sunscreen formulations.^61–63^ Our finding that alkali metal binding to AVB promotes the route to molecular dissociation is concerning given that a sunscreen mixture can come into contact with alkali metal cations, either from within the sunscreen formulation,^62^ or during contact with salts on human skin or from the sea. Given the importance of sunscreens for human health, this issue merits further investigation, potentially via advanced laser spectroscopy analysis of tailored mixtures.^35^

As discussed above, recent studies have shown that metal-ion coordination can impact on photophysical properties, a new photochemical paradigm.^9–11^ The work presented herein builds on these initial identifications of how metal-ion coordination can tune photophysical properties. Our case is, however, distinct from the two previous studies where the metal cation has strongly perturbed individual excited states.^10,11^ It is also distinct from a related study by Berenbeim *et al*. where the metal ion cation physically blocks the ultrafast vibronic decay pathways for the molecular excited states of oxybenzone.^47^ The current study instead demonstrates that electrostatic metal ion coordination in the ground electronic state can significantly alter the photochemistry of a β-diketone molecular system by modifying the ground-state tautomer population and hence the pathway to excited-state instability. This result is important in the context of the growing body of examples exploiting the potential of electric fields to exert chemical control.^64–68^

## Supplementary Material



## Notes

Methods, quantum chemical calculations, simulated IR spectra, IRMPD spectral assignment, dissociation mechanisms, discussion of the nature of the excited states, and calculations of the vertical excitation energies, oscillator strengths and character of AVB and M^+^·AVB complexes (PDF)
