# Supplementary material for: Disrupting the Photochemical Landscape of a β‑Diketone via Electrostatic Perturbation of Ground-State Tautomers
Source: J Am Chem Soc. 2025 Dec 2;147(50):45999–6007. doi: 10.1021/jacs.5c12521 (PMC12715789; doi:10.1021/jacs.5c12521)
Supplement: Supplementary file 1 [file ja5c12521_si_001.pdf]

Supplementary Material

Disrupting the Photochemical Landscape of a β-Diketone via Electrostatic Perturbation of Ground-State Tautomers

Cate S. Anstöter,[a,b†] Sarah A. Wilson,[a†] Natalie G. K. Wong,[a] Giel Berden,[c] Jos Oomens,[c] Anouk M. Rijs,[d] and Caroline E. H. Dessent[a]*

[a] Department of Chemistry, University of York, Heslington, York, YO10 5DD, UK.

[b] School of Chemistry, University of Glasgow, Joseph Black Building, University Ave, Glasgow G12 8QQ, UK.

[c] *FELIX Laboratory, Institute for Molecules and Materials, Radboud University, Toernooiveld 7, 6525 ED Nijmegen, The Netherlands.*

[d] i: Division of Bioanalytical Chemistry, Department of Chemistry and Pharmaceutical Sciences, AIMMS Amsterdam Institute of Molecular and Life Sciences, Vrije Universiteit Amsterdam, 1081 HV Amsterdam, The Netherlands: ii: Centre for Analytical Sciences Amsterdam, 1098 XH Amsterdam, The Netherlands.

† These authors contributed equally to this work.

* Corresponding author: E-mail: caroline.dessent@york.ac.uk

S1. Methods

S2. Quantum chemical calculations of the geometric structures of the complexes

S3. Simulated IR spectra of the $M^+\cdot$AVB complexes

S4. Photofragment assignments for the $M^+\cdot$AVB complexes

S5. Further details on the photofragmentation of $M^+\cdot$AVB

S6. Calculations of the Excited States of Avobenzone (AVB) and the $M^+\cdot$AVB complexes

S7. Comparison of calculated vertical excitation energies $M^+\cdot$AVB complexes

S1. METHODS

S1.a Materials

Avobenzone (AVB) was purchased from Alfa Aesar (Ward Hill, MA, USA). Solvents employed were HPLC-grade ethanol from Fisher Scientific, Inc. (Pittsburgh, PA, USA). Li, K and Rb cations were introduced as their chloride salts purchased from Fisher Scientific, Inc. (Pittsburgh, PA, USA).

S1.b IRMPD Spectroscopy

IR experiments were performed in a modified commercial quadrupole ion-trap mass spectrometer (Bruker, AmaZon Speed ETD). Solutions of AVB (1×10^{-6} mol dm^{-3}) and M^+ (1×10^{-6} mol dm^{-3}) in methanol were introduced at 180 µL/h flow rates, electrosprayed and transferred into the trap.

Ions of interest were mass selected and fragmented by IRMPD. IR spectra in the 700 - 1800 cm^{-1} region were recorded using the FELIX infrared free electron laser. FELIX was set to produce IR radiation in the form of 8-10 µs macropulses of 80 - 120 mJ per pulse at a 10 Hz repetition rate and with a bandwidth of ~0.4% of the centre frequency. The mass-selected ions were irradiated with one macropulse. Resonant absorption of IR radiation leads to an increase of the internal energy of an ion mediated by intramolecular vibrational redistribution (IVR), which eventually leads to unimolecular dissociation. After irradiation, a mass spectrum of the resulting ions in the trap is recorded. At each IR frequency point, six mass spectra were averaged. (Measured photoproducts are very low for each complex (<0.5%) and are zero for Na^+·AVB and K^+·AVB due to the expected dominant photoproduct being the M^+ ion (for Na^+ and K^+ this cannot be detected due to the ion mass falling below the low mass cut-off of the ion trap, as is described in the main manuscript on page 10. For Rb^+·AVB, ~0.5% relative intensity compared to the precursor ion IRMPD photoproducts are observed.)

The IRMPD spectra were obtained as precursor depletion spectra, using the following equation where I_p is the measured ion intensity of the precursor ion at a given wavelength: [1]

$$Precursor\ Depletion = -ln\left(\frac{I_p}{\max(I_p)}\right)$$

and plotted as a function of IR frequency. The IRMPD intensity was linearly corrected for frequency-dependent laser pulse energy. Spectra were also recorded at two levels of laser-pulse energy attenuation (factors of 2.00 and 3.16) to prevent excessive depletion of the precursor ions (saturation) and minimize formation of low *m/z* fragment ions which may be undetected in the ion trap and which would result in underestimated IRMPD intensities.

S1.c UV Photodepletion Spectroscopy

Gas-phase UV photodissociation experiments were conducted in an AmaZon SL dual funnel electrospray ionization quadrupole ion trap (ESI-QIT) mass spectrometer (Bruker Daltonics Inc., Billerica, MA, USA), modified for LIMS. Solutions of AVB (1×10^{-6} mol dm^{-3}) and M^+ (1×10^{-6} mol dm^{-3}) in ethanol were electrosprayed using typical instrumental parameters (nebulizing gas pressure of 10.0 psi, injection rate of 0.33 mL/h, drying gas flow rate of 8.0 L/min), and with capillary temperatures of 100 ◦C.

UV-Vis photons were produced by a 10 Hz Nd:YAG (Surelite™, Amplitude Laser Group, San Jose, CA, USA) pumped OPO (Horizon™, Amplitude Laser Group) laser, giving ~1.0 mJ across the range 390–238 nm. A 2 nm laser step size was used to record the photodepletion and photofragment spectra of the $M^+\cdot$AVB complexes. Photofragmentation experiments were conducted with an ion accumulation time of 20 ms and a fragmentation time of 100 ms, thereby ensuring that each mass-selected ion packet interacted with one laser pulse, minimizing the likelihood of multiphoton events. To ensure single-photon absorption conditions, the photon energy dependence on the photodepeletion signal was monitored across the spectral range. Similar measurements for the avobenzone chromophore obtained under equivalent experimental conditions are available in Ref [2].

When fluorescence is negligible, the UV excited gaseous ion will fragment upon excited-state relaxation, yielding an action absorption spectrum by photodepletion (PD). PD was measured as a function of the scanned wavelength,

$$PD = ln\left(\frac{I_{off}}{I_{on}}\right)$$

with the photofragment production (PF) also recorded simultaneously at each wavelength,

$$PF = \frac{I_{frag}}{I_{off}}$$

Where I_{off} and I_{on} are the peak intensities with laser off and on respectively, and I_{frag} is the selected fragment intensity with the laser on. PD intensities were taken from an average of three runs at each wavelength and the UV photodepletion intensity was linearly corrected for frequency-dependent laser pulse energy.

An ion-trap fragment ion cut off of *m/z* < 50 is present for both the IR or UV experiments.

S1.d Quantum chemical calculations

Enol and keto conformers of the M^+·AVB complexes, and bare AVB, were optimised using B3LYP/cc-pVTZ, with additional Stuttgart-Dresden-Bonn RLC ECP and valence basis sets for K^+ and Rb^+. All calculations were conducted for gas-phase systems. Vibrational analysis confirmed that all geometries corresponded to stationary points on the respective potential energy surfaces. Further calculations at the M062X/6-311+G**, M062X/6-311++G, and M062X/6-311G** levels of theory were conducted for the lowest-energy keto and enol isomers obtained from the B3LYP/cc-pVTZ set of optimisations, to explore the dependence of isomer ordering on computational method. Results presented are again all for stationary points on the respective potential energy surfaces.

Additional *ab initio* calculations were carried out to investigate the relative vertical excitation energies (VEEs) of the singlet and triplet states within the experimental range for the critical minimum-energy DFT conformers. These VEEs were computed using the Spin-Component Scaled Algebraic Diagrammatic Construction (SCS-ADC(2)) methodology, with the def2-SVP basis set, with the def2-ECP affixed for Rb^+. TURBOMOLE (V7.4.0) was used for these calculations.

[1] Martens, J.; Berden, G.; Gebhardt, C. R.; Oomens, J. Infrared Ion Spectroscopy in a Modified Quadrupole Ion Trap Mass Spectrometer at the FELIX Free Electron Laser Laboratory. *Rev. Sci. Instrum.* **2016**, *87* (10), 103108. doi.org/10.1063/1.4964703.

[2] Berenbeim, J.A.; Wong, N. G. K., Cockett, M.C.R.; Berden, G., Oomens, J.; Rijs, A.M.; Dessent, C. E. H. Unravelling the Keto–Enol Tautomer Dependent Photochemistry and Degradation Pathways of the Protonated UVA Filter Avobenzone, *J. Phys. Chem. A*, **2020,** *124* (15), 2919-2930. DOI: 10.1021/acs.jpca.0c01295.

S2. Quantum chemical calculations of the geometric structures of the complexes

Table S2.1 presents the relative energies and associated Boltzman populations calculated for the optimised, lowest-energy isomers of the M^+·AVB (M = Na, K, Rb) complexes, along with the relative energies for the corresponding isomers of neutral AVB.

Table S2.1: Calculated energetic and structural information for the M^+·AVB (M = Na, K, Rb) ions and neutral AVB (all gas-phase) calculated at the B3LYP/cc-pVTZ level of theory. See main text for definition of angle Φ.

	Rel. E (kJ/mol)	% Pop[a]	Isomer	Φ
Na^+·AVB	0.0	100	DK	12
	31.2	0	E1(a)	0
	36.6	0	E2(b)	0
	41.2	0	Cis-DK(b)	103
K^+·AVB	0.0	99	DK	26
	15.8	1	E1(a)	0
	19.2	0	E2(b)	0
	32.1	0	Cis-DK(b)	126
Rb^+·AVB	0.0	98	DK	32
	12.6	1	E1(a)	0
	15.6	1	E2(b)	0
	23.0	0	E2(a)	0
	24.8	0	E1(b)	0
	30.0	0	Cis-DK(b)	126
AVB	0.0	100	E1	0
	20.6	0	DK	164
	29.6	0	DK	86
	59.3	0	*Cis*-E1	140

[a] T = 375 K: Boltzmann distribution.

The results presented in Table S2.1 demonstrate unequivocally that keto-isomers are the lowest energy structures for all three M^+·AVB complexes. This is in sharp contrast with the energy ordering of uncomplexed AVB, where the E1 enol-isomer is the lowest energy structure. What is so notable about the relative isomer energies presented in Table S2.1 is the way that there is a complete reversal in the keto-enol isomer energies upon complexation, e.g. for Na^+·AVB 100% of the DK isomer compared to 100% of the E1 isomer for AVB. The results therefore

predict a complete reversal in energetic ordering of the isomers upon electrostatic complexation.

We note that the Na^+·AVB and K^+·AVB complexes do not exhibit the E1(b) or E2(a) structures observed for the Rb^+·AVB complex. This trend can likely be traced to the relative coordinate ion bond strength since for the Rb^+·AVB structures, where the ion radii are larger and inversely proportional in coordinate bond strength, so that the E1(b) and E2(a) structures can exist as stable minima.

To explore whether these results depend on the level of theory employed, further calculations were conducted using a range of functionals and basis sets i.e. B3LYP/cc-pVTZ, M062X/6-311+G**, M062X/6-311++G, and M062X/6-311G**. These combinations were chosen as they have previously been used successfully on similar alkali metal cation-molecule complexes, and in particular to interpret their IRMPD spectra.[1–4] Due to limitations of computational time, these calculations were focused on the key keto (DK) and enol (E1) isomers. For all of the calculations conducted, true geometric minima (no negative frequencies) were obtained for the complexes, and the structures were highly similar to those obtained at the B3LYP/cc-pVTZ level (Figures S2.). The relative energies for the M^+·AVB tautomers obtained from the calculations are presented in Table S2.2.

The results presented in Table S2.2 for the B3LYP/cc-pVTZ, M062X/6-311+G**, M062X/6-311++G, and M062X/6-311G** levels of theory, align well with those of Table S2.1, showing that the K-isomers of the M^+·AVB complexes are lower in energy than the E-isomers. In summary, calculations of isomeric structures of M^+·AVB lead to the conclusion that electrostatic cation binding results in the keto-isomers occurring at lower energies compared to their enol-isomer complexes, and hence reverses the isomer energy ordering for neutral AVB.

Table S2.2: Calculated relative energies for isomers of the $M^+ \cdot AVB$ complexes obtained at the B3LYP/cc-pVTZ, M062X/6-311+G**, M062X/6-311++G, and M062X/6-311G** levels of theory.[a]

		Relative Energy (kJ/mol)			
**Cation**	**Tautomer**	**B3LYP/ cc-pVTZ**	**M06-2X/ 6-311+G****	**M06-2X/ 6-311++G**	**M06-2X/ 6-311G****
**Na^+**	**Keto (DK)**	0	0	0	0
	**Enol (E1)**	31.03	32.88	32.094	35.99
**K^+**	**Keto (DK)**	0	0	0	0
	**Enol (E1)**	15.27	24.26	20.29	25.845
**Rb^+**	**Keto (DK)**	0	-	0	-
	**Enol (E1)**	11.93		16.81	

[a]. Calculations were not conducted at the M06-2X/6-311+G** and M06-2X/6-311G** levels for $Rb^+ \cdot AVB$ due to the high computational cost for Rb^+ with the diffuse functions.

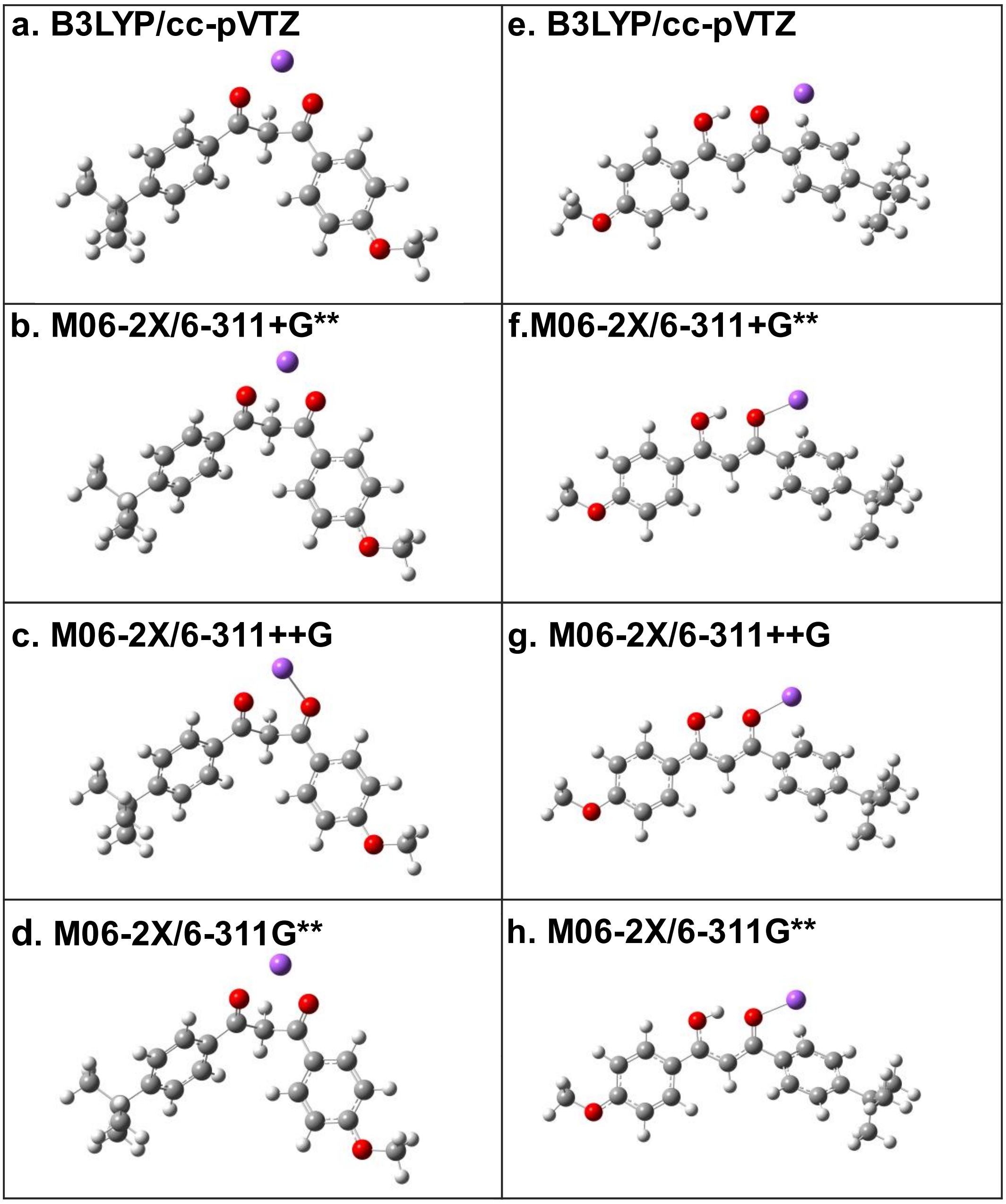

Figure S1: Optimised isomer structures for Na^{+}·AVB at the B3LYP/cc-pVTZ, M062X/6-311+G**, M062X/6-311++G, and M062X/6-311G** levels of theory: (a) Keto B3LYP/cc-pVTZ, (b) Keto/M062X/6-311+G**, (c) Keto/M062X/6-311++G (d) Keto/M062X/6-311G, (e) Enol/B3LYP/cc-pVTZ (f) Enol/M062X/6-311+G**, (g) Enol/M062X/6-311++G, (h) Enol/ M062X/6-311G**, where keto refers to DK and enol to E1 (Table S2.1).

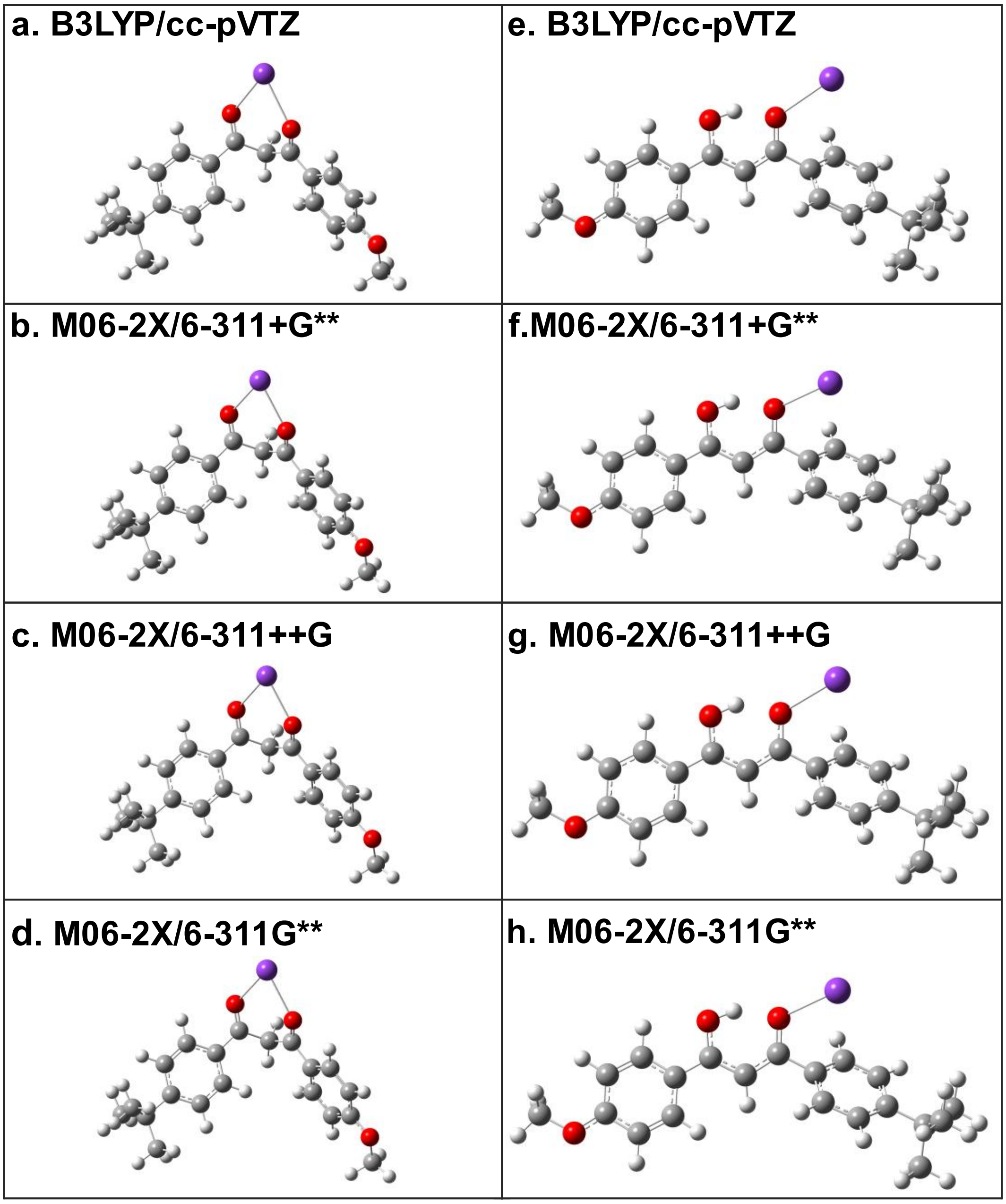

Figure S2: Optimised isomer structures for K^{+}·AVB at the B3LYP/cc-pVTZ, M062X/6-311+G**, M062X/6-311++G, and M062X/6-311G** levels of theory: (a) Keto B3LYP/cc-pVTZ, (b) Keto/M062X/6-311+G**, (c) Keto/M062X/6-311++G (d) Keto/M062X/6-311G, (e) Enol/B3LYP/cc-pVTZ (f) Enol/M062X/6-311+G**, (g) Enol/M062X/6-311++G, (h) Enol/M062X/6-311G**, where Keto refers to DK and Enol to E1 (Table S2.1).

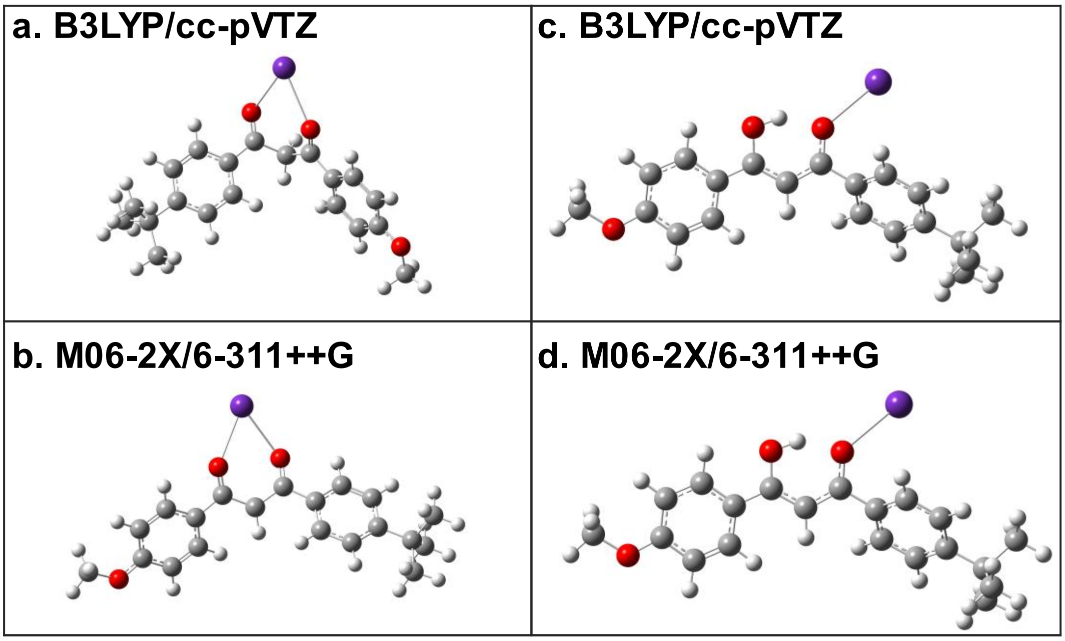

Figure S3: Optimised isomer structures for Rb^{+}·AVB at the B3LYP/cc-pVTZ, and M062X/6-311++G levels of theory: (a) Keto/B3LYP/cc-pVTZ, (b) Keto/M062X/6-311++G, (c) Enol/B3LYP/cc-pVTZ and (d) Enol/M062X/6-311++G, where Keto refers to DK and Enol to E1 (Table S2.1).

(1) Armentrout, P. B.; Boles, G. C.; Ghiassee, M.; Berden, G.; Oomens, J. Infrared Multiple-Photon Dissociation Spectra of Sodiated Complexes of the Aliphatic Amino Acids. *J. Phys. Chem. A* **2021**, *125* (29), 6348–6355. https://doi.org/10.1021/acs.jpca.1c04708.

(2) Armentrout, P. B.; Armentrout, E. I.; Clark, A. A.; Cooper, T. E.; Stennett, E. M. S.; Carl, D. R. An Experimental and Theoretical Study of Alkali Metal Cation Interactions with Cysteine. *J. Phys. Chem. B* **2010**, *114* (11), 3927–3937. https://doi.org/10.1021/jp911219u.

(3) Müller, D.; Dopfer, O. Interaction of Alkali Ions with Flavins: Infrared and Optical Spectra of Metal–Riboflavin Complexes. *J. Phys. Chem. A* **2021**, *125* (15), 3146–3158. https://doi.org/10.1021/acs.jpca.1c01846.

(4) Günther, A.; Nieto, P.; Berden, G.; Oomens, J.; Dopfer, O. IRMPD Spectroscopy of Metalated Flavins: Structure and Bonding of Mq+–Lumichrome Complexes (Mq+ = Li+–Cs+, Ag+, Mg2+). *Phys. Chem. Chem. Phys.* **2014**, *16* (27), 14161–14171. https://doi.org/10.1039/C4CP01524J.

S3. Simulated IR spectra of the $M^{+}\cdot$AVB complexes and vibrational assignment

The harmonic vibrational frequencies obtained from the calculations described in Section S2 have been used to interpret and assign the experimental IRMPD. As noted above, our choice of levels of theory was led by these methods having been successfully used previously to assign and interpret the IRMPD spectra of alkali metal cation complexes bound to molecules with carbonyls and OH groups.[1,2] B3LYP/cc-pVTZ, in particular, was found to perform well for predicting IRMPD spectra for these similar systems. Given the good previous performance of B3LYP/cc-pVTZ, we have used our current B3LYP/cc-pVTZ calculations to understand the nature of the dominant predicted vibrations. Tables S3.1 and S3.2 provide a list of the most intense vibrations predicted over the 900-1660 cm^{-1} region for the primary keto- and enol-isomers of $Na^{+}\cdot$AVB, along with mode descriptions obtained by inspection of the vibrations in GaussView.

Table S3.1: Description of vibrational modes associated with calculated peaks for the keto (DK) isomer of Na^{+}·AVB at the B3LYP/cc-pVTZ level of theory.

Calculated peak position (cm^{-1}) *	Experimental peak position (cm^{-1})	Mode description
1649 (s)	1665	Symmetric di-keto C=O stretch
1619 (w)	1635	Asymmetric di-keto C=O stretch
1597 (s)	1591	Symmetric di-keto C=O stretch coupled to C=C and C-C aromatic stretches
1579 (s)	1566	Asymmetric di-keto C=O stretch coupled to C=C and C-C aromatic stretches
1325 (s)	1324	Asymmetric C-H bend on bridging CH_2
1291 (s)	1305	C=O stretch on Anisole group and asymmetric C-H bend on bridging CH_2
1281 (m)	1282	C=O stretch on Anisole group and symmetric C-H bend on bridging CH_2
1172 (m) and 1149 (m)	1177	Asymmetric C-H bend on bridging CH_2 and aromatics
1101 (w)	1110	Asymmetric C-H bend on bridging CH_2 and torsion on aromatics
982 (m)	1018	C-H torsion on bridging CH_2 and aromatics

* The calculated frequencies are scaled by 0.97

Table S3.2: Description of vibrational modes associated with calculated peaks for the enol (E) isomer of Na^+·AVB at the B3LYP/cc-pVTZ level of theory.

**Calculated peak position (cm^{-1}) ***	**Experimental peak position (cm^{-1})**	**Mode description**
1590 (s)	1591	O-H bend on enol group
1464 (s)	n/a	O-H bend on enol group and C-H bend bridging CH out of plane
1268 (s)	1282	C=O stretch and O-H bend on enol group
1174 (s)	1177	C=O stretch on anisole group, C-H bend on bridging CH and asymmetric bend on aromatic
1017 (w)	1018	C-O stretch on anisole group CH and torsion on aromatic

Figure S4 displays the IRMPD total ion yield spectra of the Na^+·AVB complex (green) compared to calculated peaks, for both the keto (a) and enol (b) isomers. Inspection of the calculated and experimental data leads us to conclude that the keto isomer of Na^+·AVB is present experimentally. The key vibrational feature that leads us to this assignment is the peak at 1649 cm^{-1}, which is associated with the symmetric diketo carbonyl stretch. This mode is unique to the keto isomer, and can be seen clearly at 1665 cm^{-1} in the experimental spectrum. Once this peak is assigned, assignment of the experimental peaks at 1591 and 1566 cm^{-1} follows as resulting from the 1597 and 1579 cm^{-1} calculated bands associated with the symmetric and asymmetric di-keto C=O stretch coupled to C=C and C-C aromatic vibrations. In contrast, the predicted spectrum for the enol isomer of Na^+·AVB (Figure S4) has a key vibration at 1464 cm^{-1}, associated with an in-plane bend of the enol OH group. This mode is predicted to occur with a similar intensity to the 1590, 1268, and 1174 cm^{-1} vibrations, however, there are no prominent experiment peaks in the region between 1350-1550 cm^{-1}. This analysis leads us to conclude that the calculated spectrum is most consistent with the presence of a keto-isomer of Na^+·AVB.

Given this assignment, it is now possible to assign the prominent peaks at 1305, 1282 and 1177 cm^{-1} in the experimental spectrum to the 1291, 1281 and 1172 calculated modes, which are associated with C=O stretch on Anisole group and C-H bends on bridging CH_2.

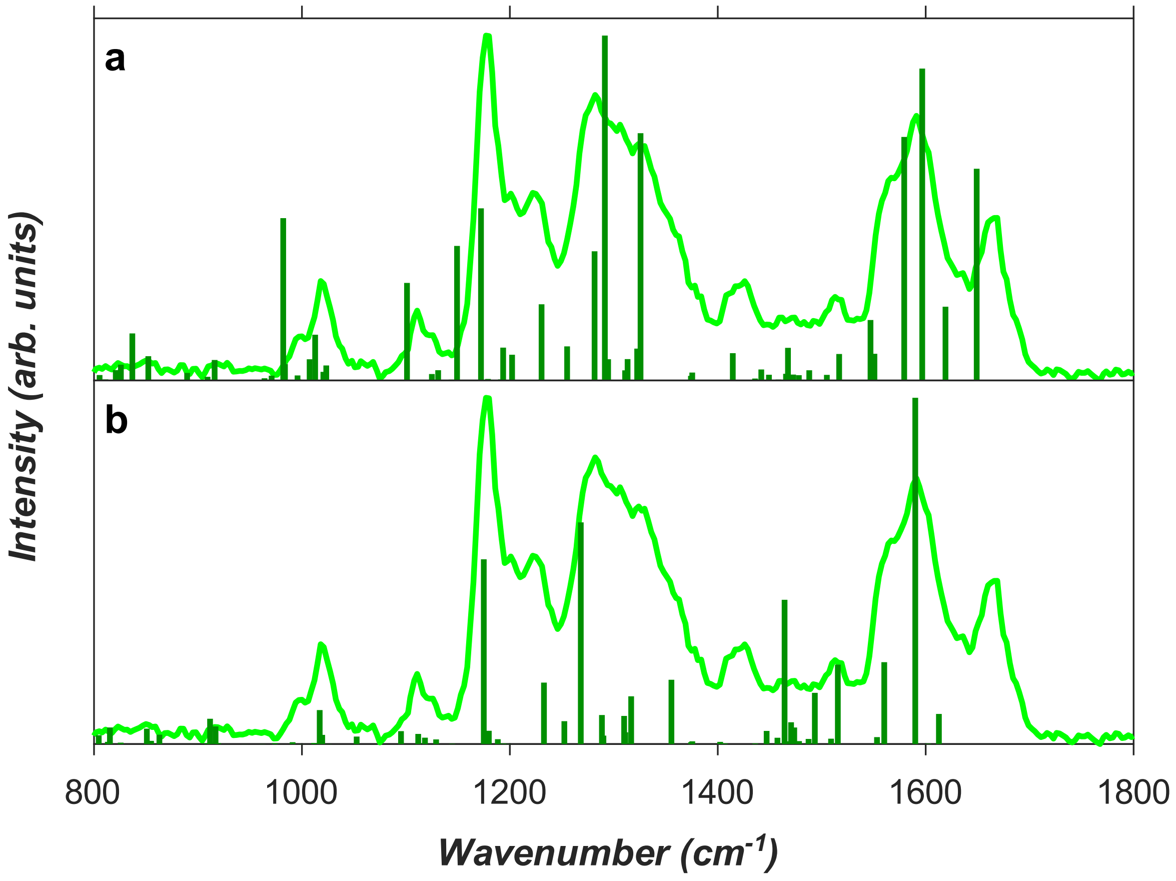

Figure S4: IRMPD spectrum of Na^+·AVB (in green) is shown overlaid with simulated spectra (in dark green) for (a) K and (b) E1. The calculated frequencies are scaled by 0.97.

Figures S5 and S6 similarly display the experimental spectra for K^+·AVB and Rb^+·AVB, along with the calculated spectra for the respective keto and enol forms. Comparison of the experimental and calculated spectra again leads us to assign the experimental spectra, primarily based on the presence of a strong feature in the experimental spectra at 1665 and 1668 cm^{-1}, respectively for K^+·AVB and Rb^+·AVB which is assigned to the symmetric diketo carbonyl stretch. Notably, this vibration is calculated to occur at 1649, 1652, and 1656 cm^{-1}, for Na^+·AVB, K^+·AVB, and Rb^+·AVB which is in good qualitative agreement with the

experimental peak values of 1665, 1666 and 1669 cm^{-1}. Tables S3.3-S3.6 provide the descriptions of the calculated vibrational modes and peak energies for the isomers of K^+·AVB and Rb^+·AVB.

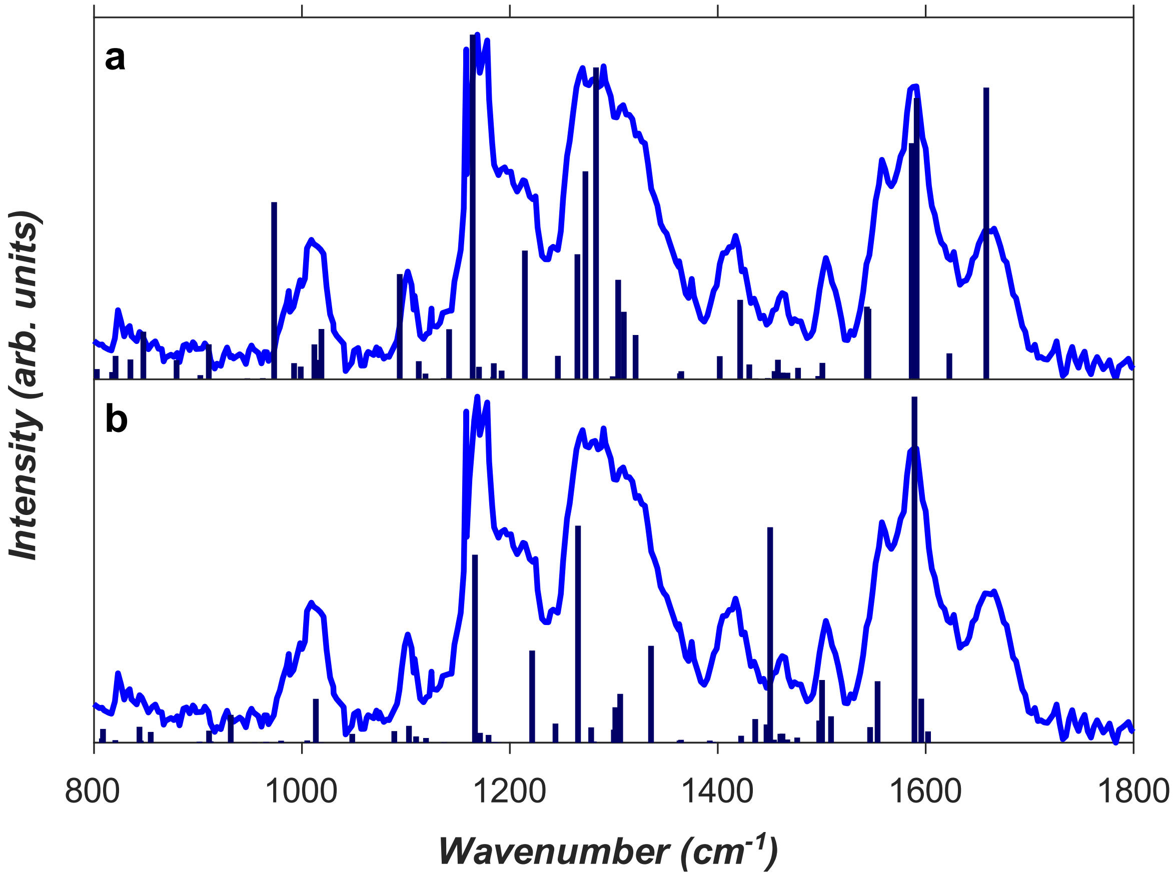

Figure S5: IRMPD spectrum of K^+·AVB (in blue) is shown overlaid with simulated spectra (in dark blue) for (a) K and (b) E1. The calculated frequencies are scaled by 0.97.

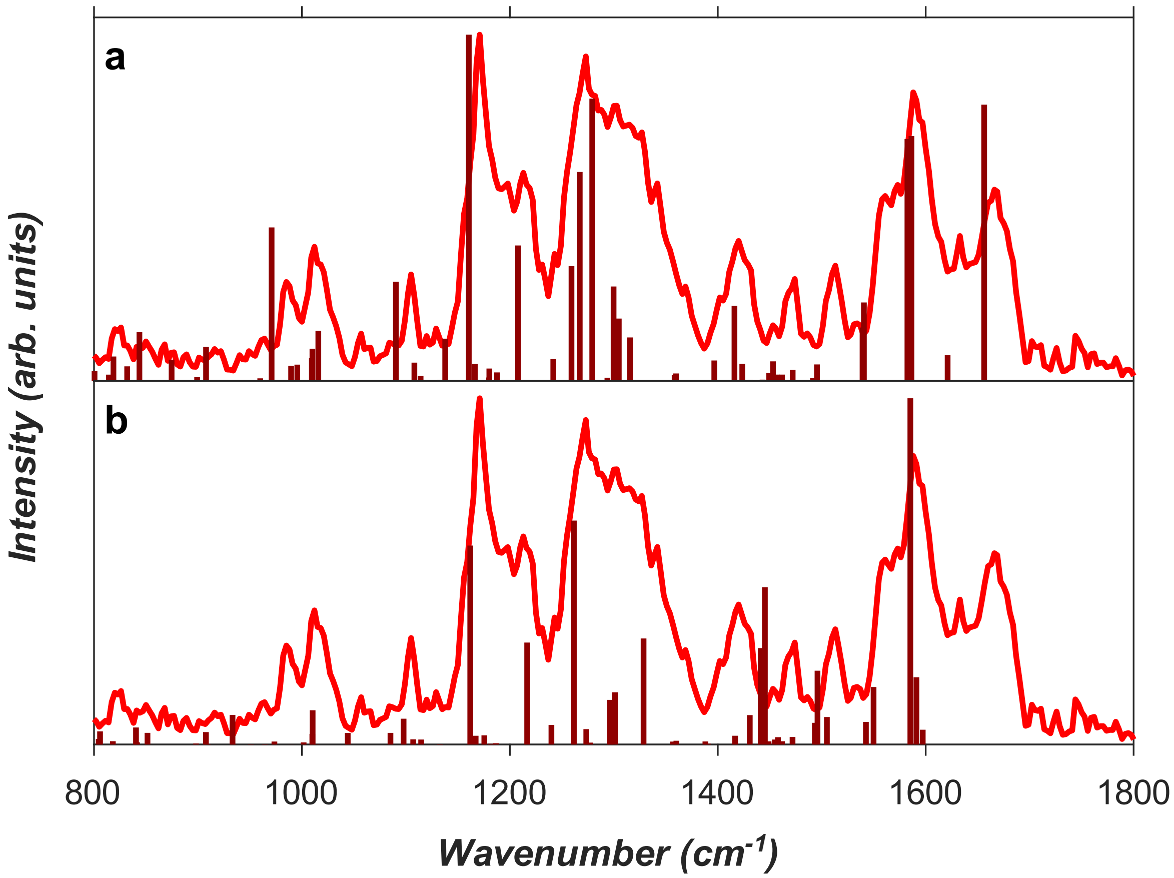

Figure S6: IRMPD spectrum of $K^{+}\cdot$AVB (in red) is shown overlaid with simulated spectra (in dark red) for (a) K and (b) E1. The calculated frequencies are scaled by 0.97.

Table S3.3: Description of vibrational modes associated with calculated peaks for the keto (DK) isomer of K^+·AVB at the B3LYP/cc-pVTZ level of theory.

**Calculated peak position (cm^{-1}) ***	**Experimental peak position (cm^{-1})**	**Mode description**
1652 (s)	1665	Symmetric di-keto C=O stretch
1617 (w)	1627	Asymmetric di-keto C=O stretch
1586 (s)	1587	Symmetric di-keto C=O stretch coupled to C=C and C-C aromatic stretches
1581 (s)	1579	Asymmetric di-keto C=O stretch coupled to C=C and C-C aromatic stretches
1278 (s)	1290	Asymmetric C-H bend on bridging CH_2
1268 (m)	1282	C=O stretch on Anisole group and asymmetric C-H bend on bridging CH_2
1260 (m)	1270	Symmetric C-H bend on bridging CH_2 and aromatics
1210 (m)	1213	Symmetric C-H bend on bridging CH_2
1160 (s)	1168	C-H bend on bridging CH_2 and aromatics
1090 (w)	1101	C-H bend on bridging CH_2 and torsion on aromatics
970 (w)	1009	C-H torsion on bridging CH_2 and aromatics

Table S3.4: Description of vibrational modes associated with calculated peaks for the enol (E) isomer of K^+·AVB at the B3LYP/cc-pVTZ level of theory.

**Calculated peak position (cm^{-1}) ***	**Experimental peak position (cm^{-1})**	**Mode description**
1583 (s)	1591	O-H bend on enol group
1445 (s)	n/a	O-H bend on enol group and C-H bend bridging CH out of plane
1261 (s)	1270	C=O stretch on anisole group, C-H bend on bridging CH and symmetric bend on aromatic
1162 (s)	1168	C=O stretch on anisole group, C-H bend on bridging CH and asymmetric bend on aromatic
1009 (w)	1011	C-O stretch on anisole group CH and torsion on aromatic

Table S3.5: Description of vibrational modes associated with calculated peaks for the keto (DK) isomer of Rb^+·AVB at the B3LYP/cc-pVTZ level of theory.

**Calculated peak position (cm^{-1}) ***	**Experimental peak position (cm^{-1})**	**Mode description**
1656 (s)	1668	Symmetric di-keto C=O stretch
1621 (w)	1632	Asymmetric di-keto C=O stretch
1586 (s)	1596	Symmetric di-keto C=O stretch coupled to C=C and C-C aromatic stretches

1582 (s)	1588	Asymmetric di-keto C=O stretch coupled to C=C and C-C aromatic stretches
1279 (s)	1288	Asymmetric C-H bend on bridging CH_2
1267 (m)	1282	C=O stretch on Anisole group and C-H bend on bridging CH_2
1259 (m)	1273	C-H bend on bridging CH_2
1207 (m)	1213	Symmetric C-H bend on bridging CH_2
1160 (s)	1171	C=O stretch on anisole group and C-H bend on aromatics
1090 (w)	1104	Asymmetric C-H bend on bridging CH_2 and aromatics
970 (m)	985	C-H torsion on bridging CH_2 and aromatics

Table S3.6: Description of vibrational modes associated with calculated peaks for the enol (E) isomer of Rb^+·AVB at the B3LYP/cc-pVTZ level of theory.

**Calculated peak position (cm^{-1}) ***	**Experimental peak position (cm^{-1})**	**Mode description**
1585 (s)	1588	O-H bend on enol group
1495 (w)	n/a	C=O stretch on enol group
1445 (s/m)	n/a	O-H bend on enol group and C-H bend on bridging CH out of plane
1328 (m)	1342	C=O and C-O stretch on enol group
1261 (s)	1273	O-H bend on enol group, C=O stretch on anisole group, C-H

		bend on bridging CH and symmetric bend on aromatic
1162 (s)	1171	C=O stretch on anisole group, C-H bend on bridging CH and asymmetric bend on aromatic
1010 (w)	1012	C-O stretch on anisole group CH and torsion on aromatic

Finally, Table S3.7 provides an assignment of the vibrational features observed in the experimental spectra, following the mode descriptions of Tables S3.1, S3.3 and S3.5 for the enol isomers.

[1] Armentrout, P. B.; Boles, G. C.; Ghiassee, M.; Berden, G.; Oomens, J. Infrared Multiple-Photon Dissociation Spectra of Sodiated Complexes of the Aliphatic Amino Acids. J. Phys. Chem. A 2021, 125 (29), 6348–6355. https://doi.org/10.1021/acs.jpca.1c04708.

[2] Günther, A.; Nieto, P.; Berden, G.; Oomens, J.; Dopfer, O. IRMPD Spectroscopy of Metalated Flavins: Structure and Bonding of Mq+–Lumichrome Complexes (Mq+ = Li+–Cs+, Ag+, Mg2+). *Phys. Chem. Chem. Phys.* **2014**, *16* (27), 14161–14171. https://doi.org/10.1039/C4CP01524J.

S4. Photofragment assignments for the M^{+}·AVB complexes

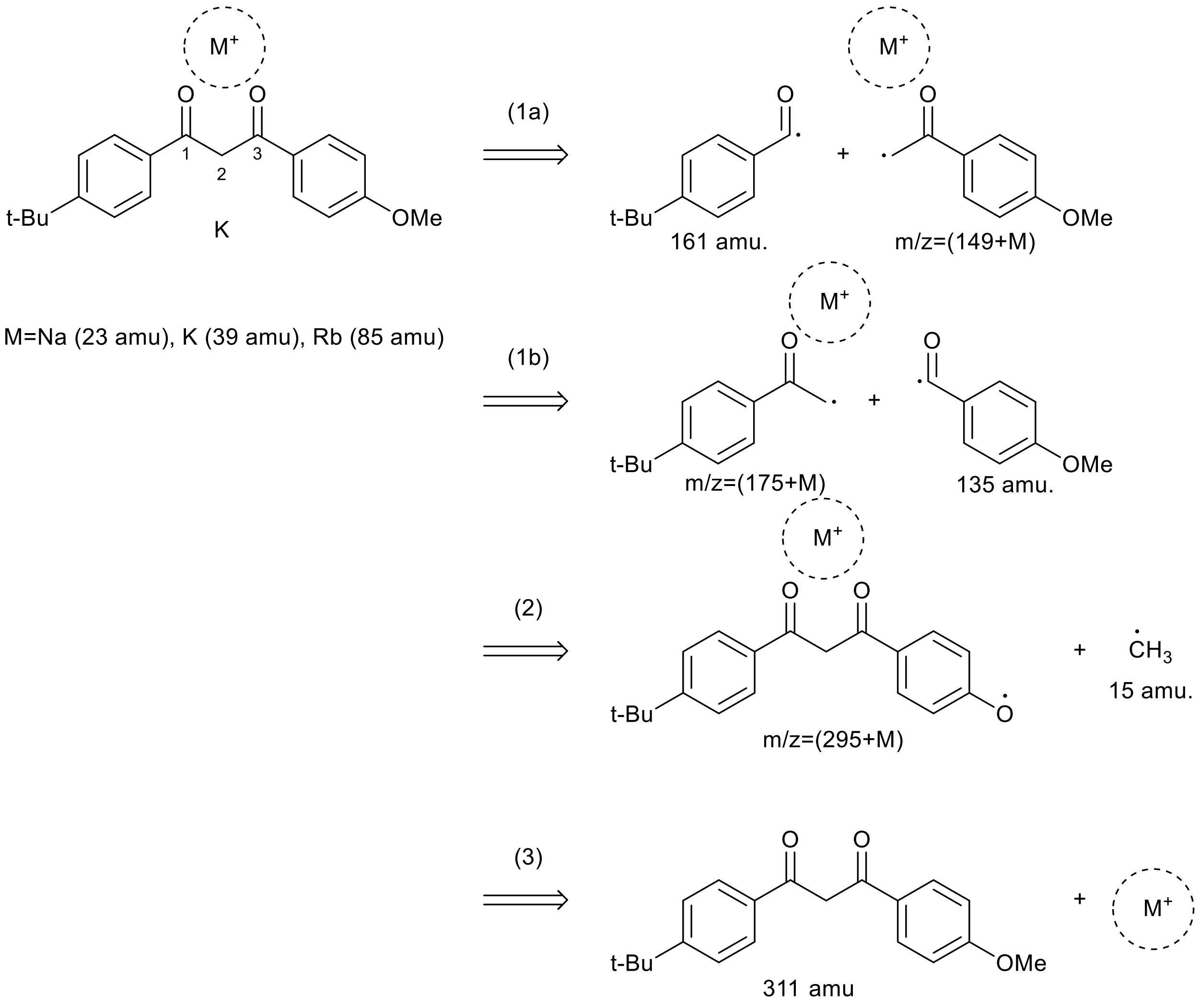

Figure S7: Proposed photofragmentation products (α-cleavage (1a, 1b), homolytic OMe dissociation (2), and cation loss (3)) of M^{+}·AVB. Pathways 1-3 were observed following UV excitation, while only fragmentation pathway (3) occurred upon IRMPD.

S5. Further details on the photofragmentation of M^+·AVB

For Na^+·AVB, the photofragments (PFs) observed were *m/z* 172, 198 and 318, corresponding to fragmentation pathways [1a], [1b] and [2], respectively. (Pathways are illustrated in Section S4) The *m/z* 318 ion associated with homolytic CH_3-O rupture and methyl loss (Pathway [2]) was observed to be the dominant PF, with the other two PFs, *m/z* 172 and 198, appearing with intensities at least four times lower than *m/z* 318. All three PFs displayed a peak in production around 4.0 eV, with *m/z* 318 being produced strongly through the 4.70 eV peak.

The PFs observed for K^+·AVB, were *m/z* 188, 214 and 334 PFs, which like Na^+·AVB correlated with fragmentation pathways [1a], [1b] and [2]. In contrast to Na^+·AVB, the K^+·AVB PFs all displayed similar production intensities across the full excitation region. Peaks in PF production were visible at 4.30 and 4.90 eV.

For Rb^+·AVB, the PFs observed were *m/z* 85, 234, 260, and 380. As for the Na^+·AVB and K^+·AVB complexes, the *m/z* 234, 260, and 380 correlated with fragmentation pathways [1a], [1b] and [2]. The m/z 85 PF corresponds to Rb^+ loss, as depicted in pathway [3].

For interpreting the nature of the M^+·AVB excited states observed in the photodissociation experiments, it is useful to measure the collision-induced dissociation pathways for the system under study to allow us to understand if excited-state dissociation is statistical or non-statistical. Collision-induced dissociation of M^+·AVB is expected to result in cluster fission as in other simple ion-molecule clusters, with ejection of M^+ *i.e.* pathway [3]. We do indeed observe this for Rb^+·AVB, where the Rb^+ mass is above the trap low-mass cut off, and it is reasonable to assume similar CID behavior for the other complexes. (These complexes decay upon CID at with dissociation onsets of 25%, 12% and 0% HCD Energy, for Na^+·AVB, K^+·AVB and Rb^+·AVB, respectively. These values align with the expected trend for the strength of ion binding order, i.e. $Na^+ > K^+ > Rb^+$.)

Photoinitiated production of the M^+ cation (observation of pathway [3]), which matches the electronic ground state CID pathway, would be associated with a statistical decay process for an excited state. [1] Conversely, the AVB rupture pathways [1]-[2] would be associated with non-statistical (photochemical excited state) decay pathways. The excited states present at

lower photoexcitation energies therefore appear to display a greater degree of statistical decay behavior whereas those at the higher photoexcitation energies (>4 eV) display a greater propensity for non-statistical decay.

Reference

1. Cercola, R.; Matthews, E.; Dessent, C. E. H. Photoexcitation of Adenosine 5′-Triphosphate Anions in Vacuo: Probing the Influence of Charge State on the UV Photophysics of Adenine. *J. Phys. Chem. B* **2017**, *121* (22), 5553–5561. https://doi.org/10.1021/acs.jpcb.7b03435.

S6. Calculations of the Excited States of Avobenzone (AVB) and the M^{+}·AVB complexes

AVB

Table S6.1: SCS-ADC(2) calculated vertical excitation energies (eV), oscillator strengths and dominant character for the electronic transitions of the Enol (E) tautomer of Avobenzone (AVB)

AVB (E)	VEE / eV	Osc Strength	Dominant Char
S_1	3.674	0.006610	n->π*
S_2	3.924	0.851598	π->π*
S_3	4.740	0.013552	π->π*
S_4	4.834	0.024047	π->π* (CT)
S_5	4.868	0.196514	π->π* (CT)
S_6	5.545	0.213265	π->π*

Table S6.2: SCS-ADC(2) calculated vertical excitation energies (eV), oscillator strengths and dominant character for the electronic transitions of the Keto (K) tautomer of Avobenzone (AVB)

AVB (K)	VEE / eV	Osc Strength	Dominant Char
S_1	3.628	0.003970	n->π*
S_2	3.764	0.000544	n->π*
S_3	4.826	0.003510	π->π*
S_4	4.838	0.002943	π->π*
S_5	5.062	0.490203	π->π*
S_6	5.534	0.418675	π->π*

AVB·Na⁺

Table S6.3: SCS-ADC(2) calculated vertical excitation energies (eV), oscillator strengths and dominant character for the electronic transitions of the Enol (E) tautomer of Na^{+}·AVB

AVB·Na^{+} (E)	VEE / eV	Osc Strength	Dominant Char
**S_1**	3.572	**1.184733**	π->π*
**S_2**	4.233	0.013489	π->π*
**S_3**	4.598	0.181496	π->π* (CT)
**S_4**	4.751	0.014353	π->π*
**S_5**	4.891	0.026395	π->π*
**S_6**	5.060	0.007982	n->π*

Table S6.4: SCS-ADC(2) calculated vertical excitation energies (eV), oscillator strengths and dominant character for the electronic transitions of the Keto (K) tautomer of Na^{+}·AVB

AVB·Na^{+} (K)	VEE / eV	Osc Strength	Dominant Char
**S_1**	4.131	0.111429	n->π*
**S_2**	4.227	**0.352395**	π->π* (CT)
**S_3**	4.367	0.095974	n->π*
**S_4**	4.520	0.020196	π->π* (CT)
**S_5**	4.686	0.000094	π->π* (CT)
**S_6**	4.788	**0.490523**	π->π* (CT)

AVB·K^+

Table S6.5: SCS-ADC(2) calculated vertical excitation energies (eV), oscillator strengths and dominant character for the electronic transitions of the Enol (E) tautomer of K^+·AVB

AVB·K^+ (E)	VEE / eV	Osc Strength	Dominant Char
S_1	3.69124	1.086513	π->π*
S_2	4.40833	0.011169	n->π*
S_3	4.46908	0.023442	π->π*
S_4	4.74394	0.101131	π->π*
S_5	4.81276	0.091625	π->π*
S_6	5.03659	0.087822	π->π*

Table S6.6: SCS-ADC(2) calculated vertical excitation energies (eV), oscillator strengths and dominant character for the electronic transitions of the Keto (K) tautomer of K^+·AVB

AVB·K^+ (K)	VEE / eV	Osc Strength	Dominant Char
S_1	4.129	0.011567	n->π*
S_2	4.234	0.036756	n->π*
S_3	4.449	0.494258	π->π*
S_4	4.613	0.011266	π->π*
S_5	4.739	0.000232	π->π* (CT)
S_6	4.948	0.502452	π->π*

AVB·Rb^+

Table S6.7: SCS-ADC(2) calculated vertical excitation energies (eV), oscillator strengths and dominant character for the electronic transitions of the Enol (E) tautomer of Rb^+·AVB

AVB·Rb^+ (E)	VEE / eV	Osc Strength	Dominant Char
S_1	3.710	1.082358	π->π*
S_2	4.373	0.010811	n->π*
S_3	4.496	0.022499	π->π* (CT)
S_4	4.736	0.116520	π->π*
S_5	4.802	0.077608	π->π* (CT)
S_6	5.068	0.092619	π->π*

Table S6.7: SCS-ADC(2) calculated vertical excitation energies (eV), oscillator strengths and dominant character for the electronic transitions of the Keto (K) tautomer of Rb^+·AVB

AVB·Rb^+ (K)	VEE / eV	Osc Strength	Dominant Char
S_1	4.156	0.003086	n->π*
S_2	4.225	0.016920	n->π*
S_3	4.502	0.543945	π->π* (CT)
S_4	4.642	0.009339	π->π*
S_5	4.751	0.000266	π->π*
S_6	5.005	0.495337	π->π*

S7. Comparison of calculated Vertical excitation energies M^+·AVB complexes

The data shown on Figure S8 illustrate how there are only modest differences in the VEEs for the key excited states of the three M^+·AVB complexes, so that changing the metal cation only results in minor perturbations of the relative locations of the excited states. For example, the VEEs for both the $1\pi\pi^*$ and $2\pi\pi^*$ states of the ketones increase modestly on going from Na^+·AVB to Rb^+·AVB.

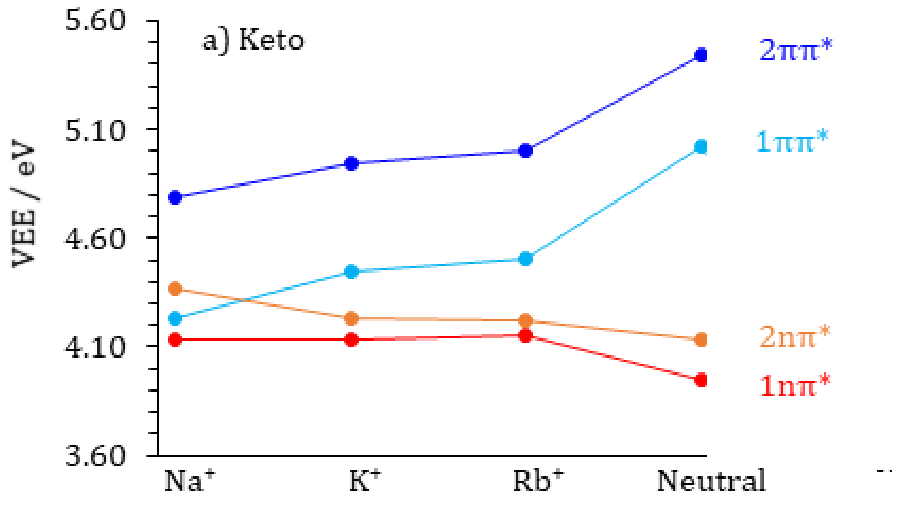

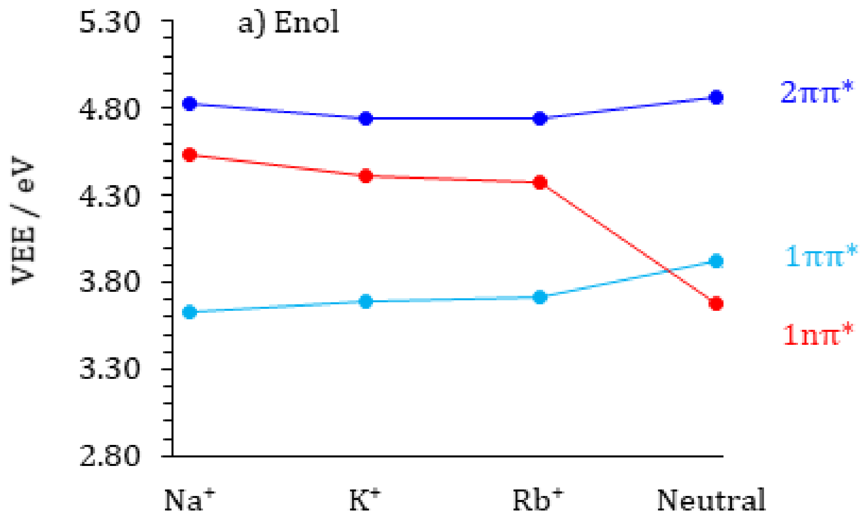

Figure S8: Calculated vertical excitation energies (eV) over the range 2.80-5.50 eV for the bright electronic transitions of the M^+·AVB complexes, along with neutral and protonated AVB, for their a) keto and b) enol tautomers.
